# Communal nesting is the optimal strategy for heat conservation in a social marsupial: lessons from biophysical models

**DOI:** 10.1242/jeb.244606

**Published:** 2022-11-25

**Authors:** Roberto F. Nespolo, Isabella Peña, Carlos Mejías, Abel Ñunque, Tomás Altamirano, Francisco F. Bozinovic

**Affiliations:** ^1^ Instituto de Ciencias Ambientales y Evolutivas, Universidad Austral de Chile, Valdivia, Chile; ^2^ Facultad de Ciencias, Universidad Austral de Chile, Valdivia, Chile; ^3^ Center of Applied Ecology and Sustainability (CAPES), Pontificia Universidad Católica de Chile, Santiago, Chile; ^4^ Millennium Nucleus of Patagonian Limit of Life (LiLi), Valdivia, Chile; ^5^ Magister en Ecología Aplicada, Facultad de Ciencias, Universidad Austral de Chile, Valdivia, Chile; ^6^ ECOS (Ecology-Complexity-Society) Laboratory, Center for Local Development (CEDEL), Pontificia Universidad Católica de Chile, Villarrica Campus, La Araucanía Region, Chile; ^7^ National Audubon Society and Cape Horn International Center for Global Change Studies and Biocultural Conservation, Universidad de Magallanes, Punta Arenas, Chile; ^8^ Departamento de Ecología Facultad de Ciencias Biológicas, Pontificia Universidad Católica de Chile, Santiago, Chile; ^9^ Millennium Nucleus Center for the Socioeconomic Impact of Environmental Policies (CESIEP), Chile

**Keywords:** Nest, Taxidermic models, Mannequin, Marsupial, Newton's passive cooling, Endothermy, Thermoregulation

## Abstract

Endothermy, understood as the maintenance of continuous and high body temperatures owing to the combination of metabolic heat production and an insulative cover, is severely challenged in small endotherms inhabiting cold environments. As a response, social clustering combined with nest use (=communal nesting) is a common strategy for heat conservation. To quantify the actual amount of energy that is saved by this strategy, we studied the social marsupial *Dromiciops gliroides* (monito del monte), an endemic species of the cold forests of southern South America. It is hypothesized that sociability in this marsupial was driven by cold conditions, but evidence supporting this hypothesis is unclear. Here, we used taxidermic models (‘mannequins’) to experimentally test the energetic benefits of clustering combined with nest use. To do this, we fitted and compared cooling curves of solitary and grouped mannequins, within and outside of a nest, at the typical winter ambient temperatures of their habitat (5°C). We found that the strategy that minimized euthermic cost of maintenance was the combination of nest use and clustering, thus supporting communal nesting as a social adaptation to cope with the cold. Considering the basal metabolic rate of monitos, our estimates suggest that the savings represents almost half of energy consumption per day (in resting conditions). This study shows how simple biophysical models could help to evaluate bioenergetic hypotheses for social behavior in cold-adapted endotherms.

## Teaser

**Summary:** A simple biophysical model shows that nest building and huddling represent the optimal strategies for energy savings in a cold-adapted marsupial.

## INTRODUCTION

Organisms exist in thermodynamic equilibria with their environment (Porter and Gates, 1969), and cold conditions create especially tough challenges for small, endothermic animals with high rates of heat dissipation, owing to their high surface area-to-volume ratio (Canals et al., 1989). Although fur and feathers provide good insulation, at very small sizes these are not enough to maintain sustained euthermia. Thus, additional strategies were promoted by natural selection, among which some are physiological, such as increase in thermogenic capacity and heterothermic responses (Bozinovic et al., 1987; Nespolo et al., 1999; Sharbaugh, 2001), while some others are behavioral, such as the formation of clusters or huddling, the use of nests or a combination of both (i.e. communal nesting) (Bozinovic et al., 1988; Canals et al., 1989; Canals et al., 1997; Gilbert et al., 2010; Scholander, 1955).

The fact that clustered individuals conserve heat more efficiently than isolated ones is a generalized biophysical consequence of the exponential reduction of surface-to-volume ratio (Canals et al., 1997). Thus, it is not surprising that huddling is widespread in small birds and mammals of cold environments (e.g. marsupials: Fisher et al., 2011; rodents: Antinuchi and Busch, 2001; Kauffman et al., 2003; birds: Lubbe et al., 2018; see reviews in Gilbert et al., 2010; Wojciechowski et al., 2011). However, communal nesting is also attributed to other factors, such as social behavior, protection and structural support (reviewed by Ebensperger and Labra, 2020). For instance, in the Siberian flying squirrel, clustering behavior is explained by subsequent mating rather than kinship or thermoregulating benefits (Selonen et al., 2014). The avian cup-shaped nest design is primarily explained by structural support, and not insulation (Heenan and Seymour, 2011). Similarly, in the social degu (*Octodon degus*), communal nesting is explained by kinship, rather than thermoregulation (Ebensperger and Bozinovic, 2000; Ebensperger et al., 2004). However, it has been challenging to test these competing hypotheses on live animals, either in the laboratory or in the field, owing to the obvious limitations of obtaining the animals, the precision of the technique for measuring energy consumption, or even obtaining the nests.

Here, we performed a laboratory study with biophysical models focused on the social marsupial monito del monte (*Dromiciops gliroides* Thomas 1894), which, according to several observations in both captive and free-ranging individuals, exhibits advanced levels of sociality (reviewed in Nespolo et al., 2022b). They normally cluster together, at any season, in groups that can have 10 individuals (typical cluster size is 3–4) (Franco et al., 2011; Nespolo et al., 2022a) and build sophisticated nests (Vazquez et al., 2020). Detailed descriptions of *Dromiciops* biology, physiology and life cycle were recently published (Fonturbel et al., 2022; Nespolo et al., 2022b). Briefly, *D. gliroides* is an arboreal, nocturnal small mammal (∼30 g) endemic to the temperate rainforests of southern South America, which includes high Andean locations where winter temperatures reach freezing values, but also mild locations near the coast with mean winter temperatures of approximately 5°C (Gurovich et al., 2015; Mejías et al., 2021). Two species of monito are now recognized (*D. gliroides* and *D. bozinovici*), which are intimately associated with the temperate rainforests of southern South America, an ecosystem that is patchily distributed over a latitudinal range of approximately 1000 km at both sides of the Andes range (from 35°S to 43°S). The activity cycle of monitos is highly seasonal, characterized by a hibernation period of approximately 6 months and a breeding period of 4 months, followed by a fattening stage (see details in Nespolo et al., 2022b). However, monitos are opportunistic heterotherms with variable hibernation periods depending on latitude and food availability. For instance, active animals have been observed during winter (Nespolo et al., 2022a; Oda et al., 2019), and torpid animals during breeding periods (females with pups, all torpid, see Nespolo et al., 2021).

Monitos elaborate complex spherical nests, built by interlaced quila leaves (*Chusquea quila*, an endemic bamboo), covered with moss inside, and the whole structure is usually located within tree cavities (Celis-Diez et al., 2012; Franco et al., 2011; Honorato et al., 2016; Vasquez et al., 2018). It is likely that monitos build communal nests as a heat conservation and energy saving strategy, which could explain the origin of their sociality. However, two facts complicate this conclusion. First, measurements of energy expenditure in clustered and isolated individuals did not produce significant differences (Franco et al., 2012). Second, as it is known that marsupials have a tropical origin (Mitchell et al., 2014), it could be the case that the sociality of monitos preceded the colonization of cold environments (Fonturbel et al., 2022; Nespolo et al., 2022b; Russell, 1984). Therefore, showing a net energetic benefit of communal nesting over solitary strategies would support the idea that sociability arose in part from having to face a cold environment.

Here, we performed laboratory experiments for simulating clustering, combined with nest use, and tested whether these strategies are synergistic in conferring energetic savings. If communal nesting represents an adaptive strategy to cope with the cold, then clustered individuals within nests should significantly reduce the endothermic cost of thermoregulation compared with isolated individuals without nests.

**List of symbols and abbreviations**

*C*: minimum thermal conductance
*E*_cost_: energy needed to maintain euthermic thermoregulation
half-time: time needed to reduce 50% of temperature
*k*: rate constant (reciprocal of time)
*T*_A_: ambient temperature
*T*_B_: body temperature
*y*: temperature at each time interval
*y*_0_: initial temperature
τ: reciprocal of *k*

## MATERIALS AND METHODS

**Fig. 1.**
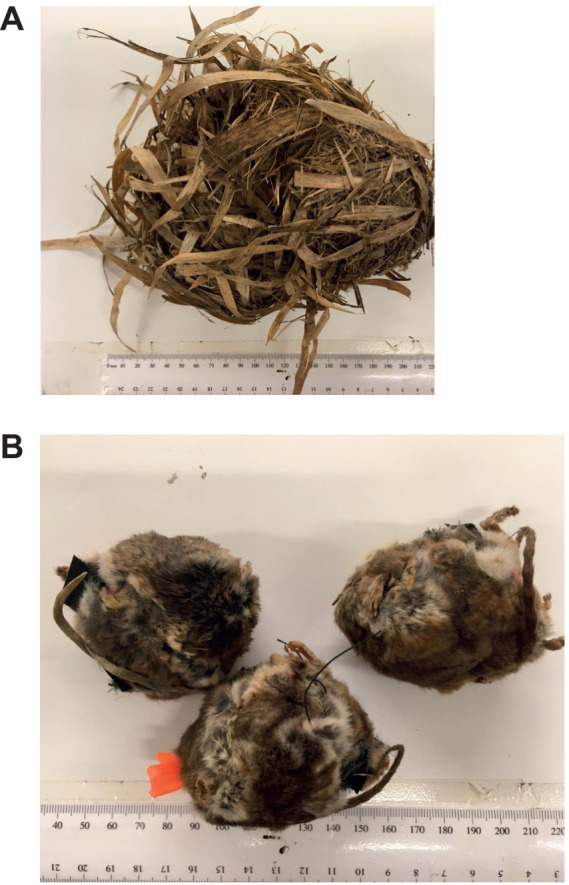
**Images of nests and mannequins.** (A) Monito nests used in this study. (B) The mannequins used for the cooling curves.

We used real monito nests obtained from a bird conservation program run by one of the authors (T.A.). We had access to 240 nesting boxes located at Pucón (39°18′51″S, 71°52′50″W, 1100 m.a.s.l.) that are normally colonized by birds in spring and monitos in autumn. During the 2019 winter, we obtained 17 monito nests (dry mass 55.1±2.4 g, mean±s.e.m.) (Fig. 1) found within nest boxes at the end of the winter. Each nest was individually placed in a paper bag and stored until the start of our experiment, 2 months later.

The taxidermic models (‘mannequins’, hereafter) were manufactured with skins of dead monitos, provided by the National Services for Wildlife (Servicio Agrícola y Ganadero and Corporación Nacional Forestal, Chile). Nine carcasses were used, which were freshly dissected and left for 2–3 days stretched, covered by a layer of sodium chloride and boron sulphate to dry out. Two skins were needed to manufacture one individual, thus we built five mannequins in total (Fig. 1). Each mannequin was fabricated using a 40 g agar bead, because it has similar thermal properties with living tissue. In the range of 5–40°C, agar has on average, a conductance of 0.56 W m^−1^ K^−1^ (Zhang et al., 2011), whereas the thermal conductance of living soft tissue (e.g. brain and abdomen) ranges from 0.53 to 0.55 W m^−1^ K^−1^ (Rough and Bharathan, 2005). The agar was covered by the skin and sutured with surgical sutures. In the middle of the agar sphere, a 4×9 mm (diameter×length) cylindrical space was left to introduce the temperature sensor (see below), and a Velcro closure was included for opening. The agar was prepared with 3 g of agar–agar and 100 ml of boiling water. The sphere was wrapped in a rubber balloon so that the agar did not dehydrate at high temperatures. The mannequin's mass was 55.1±2.4 g, with a volume of 39.8±2.7 ml and a density of 1.4±0.09 g ml^−1^. This is within the range of well-fed monitos (typical of autumn; range: 30–60 g; see Bozinovic et al., 2004; Nespolo et al., 2021).

For the cooling curves, we used two ‘Pelt’ chambers (60×40×30 cm, Sable Systems, North Las Vegas, NV, USA) to maintain constant temperatures, one for warming up the mannequins to 40±1°C and the other for cooling it at 5±1°C (the actual cooling curve). This temperature is representative of the coastal habitat of *D. gliroides*, which exhibits a winter mean temperature of 5.5°C (max.=8.1°C; min.=3.3°C) (Nespolo et al., 2022a).

We placed a wire frame in the floor of the chamber so that neither mannequins nor nests were in direct contact with the chamber material. For each cooling experiment, the procedure consisted of removing the mannequin from the warm chamber, waiting for it to cool down to 35°C (the euthermic body temperature of a monito), and placing it within the cold chamber. We performed two cooling curves per day. To continuously record the core temperature of the mannequins, we used temperature data loggers (model DST-micro, Star Oddi, Iceland; cylindrical 25 mm long and 5 mm in diameter, resolution: 0.003°C) with a data-gathering frequency of one per minute. According to the manufacturer, the devices are calibrated at the factory over a temperature range of 5 to 45°C. We also tested the devices in a beaker with water at 40°C that was allowed to cool to room temperature (10°C), with temperature recorded every 2 min, using a copper-constantan thermocouple (Cole Parmer). The linear regression between water and logger temperature (20 points) was highly significant (*R*^2^=0.99, *P*=0.001). The environmental temperature within the chambers was recorded, in addition to the chamber's thermocouple, with a HOBO environmental temperature data logger (model pro v2, Onset Computer Corporation, Bourne, MA, USA) attached to the internal walls of the chamber, with the probe hanging in the air. This probe was configured to record one datapoint per minute. Every cooling trial had a duration of 593 min, and we performed a total of 50 curves combining solitary, clustered and individuals within nests. This was an arbitrary duration decided after inspecting a few preliminary trials, which indicated that temperatures reach an asymptotic value after approximately 8 h (500 min; see Fig. 4C). We ran five curves with a bare datalogger (the data logger without agar nor fur), five curves with the agar sphere (no fur), 10 curves with a solitary mannequin (data logger with agar with fur), 10 curves with a solitary mannequin within a nest, 10 curves with a group of three mannequins and 10 curves with a group of three mannequins within a nest. For visualization (and for solely illustrative purposes), we took infrared thermographic images at different stages of the warming using a thermograph (FLIR i40, Sweden). The thermograph was located 25 cm away from the mannequins and set to an emissivity of 0.98. Images were taken at 0, 4 and 8 h in solitary mannequins, and also repeated for grouped mannequins. These were different trials than those used for the presented analyses (for which we did not open the chamber). Each cooling curve was adjusted to a one-phase exponential decay given by the equation: *y*=(*y*_0_–plateau)e^−*k*×time^+plateau, where *y* is the temperature at each time interval, *y*_0_ is *y* at time=zero, the plateau is the asymptotic temperature of the taxidermic model, and *k* is the rate constant, expressed as the reciprocal of time (the higher the *k*, the higher the heat loss rate). We also calculated the time constant (τ), as the reciprocal of *k*, and the half-time, as ln(2)/*k*.

In addition to estimating curve parameters, we predicted the euthermic cost of maintenance by calculating the energy that a monito would spend to maintain its body temperature constant (*E*_cost_), for every minute. Thus, we used Newton's cooling equation for passive cooling, which is normally used for estimating metabolic rates in steady-state conditions among endotherms (Mejías et al., 2022; Nicol and Andersen, 2007; Rezende and Bacigalupe, 2015), and calculated *E*_cost_ as: *C*(*T*_B_−*T*_A_), where *C* is the minimum thermal conductance of euthermic *D. gliroides* (*C*=3.4848 J g^−1^ h^−1^ °C^−1^) (Bozinovic et al., 2004), *T*_B_ is body temperature (°C) and *T*_A_ is ambient temperature (°C). We also calculated the overall *E*_cost_ (=net *E*_cost_) for the entire cooling curve. Curve parameters (i.e. rate constant, half-time, time constant) and net *E*_cost_ were compared among the six treatments: bare data logger, data logger with agar, data logger with fur (=mannequin), grouped mannequins (three individuals), individual mannequin within a nest and grouped mannequins within a nest. These were compared with a one-way ANOVA, using Statistica software (StatSoft, Tulsa, OK, USA). Parametric assumptions were checked using Levene’s test for homogeneity of variance and the Shapiro–Wilk test for normality.

## RESULTS

We found significant differences among treatments in rate constant (one-way ANOVA, *F*_5,43_=495.5, *P*=0.0001), half-time (*F*_5,43_=65.7, *P*=0.0001) and time constant (*F*_5,43_=65.8, *P*=0.0001; see Table 1). The agar (i.e. the difference in cooling rate of the data logger with and without agar) reduced the rate of heat loss by a factor of 12 (from 0.37±0.02 to 0.031±0.002 min^−1^, Fisher *post hoc* test, *P*=0.001; Table 1). In contrast, the fur (i.e. the difference in cooling rate of the mannequin with and without skin) reduced the rate of heat loss by a factor of 2.3 (from 0.031±0.002 to 0.014±0.0006 min^−1^, Fisher *post hoc* test, *P*=0.001; Table 1). However, the rate constant was not significantly different between solitary, grouped, solitary in nests and grouped in nests mannequins (Fisher *post hoc* tests, *P*>0.6; Table 1).

**Table 1.**
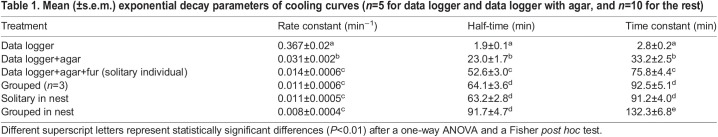
Mean (±s.e.m.) exponential decay parameters of cooling curves (*n*=5 for data logger and data logger with agar, and *n*=10 for the rest)

**Fig. 2.**
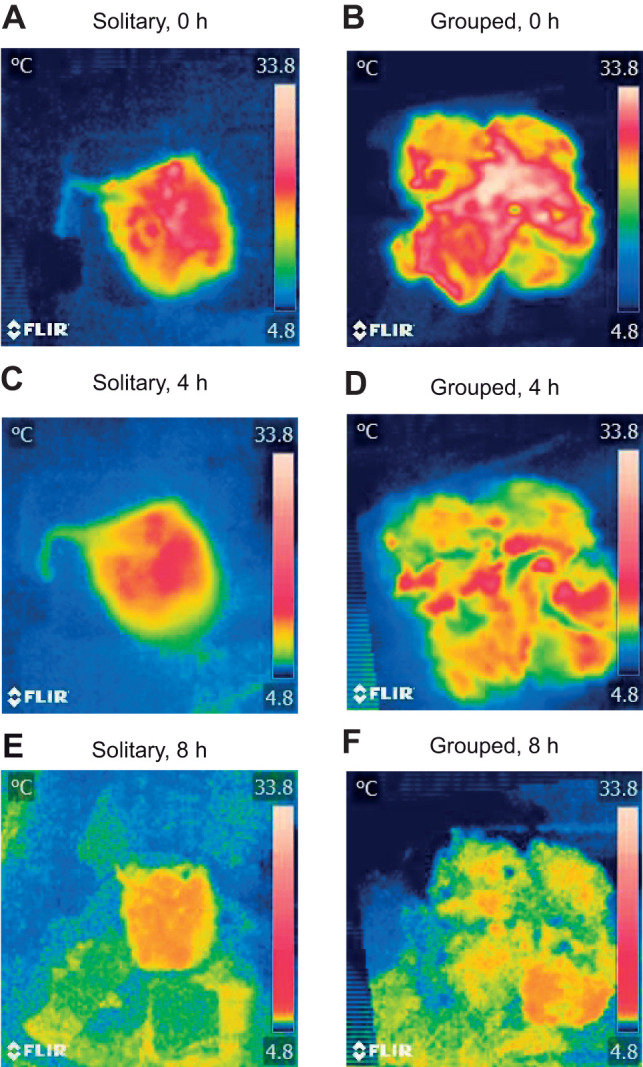
**Representative thermographic images obtained from grouped and solitary mannequins at different moments of the cooling trial.** The images show how the outermost surface of the skin with fur is colder than the core, thus acting as an isolating envelope.

**Fig. 3.**
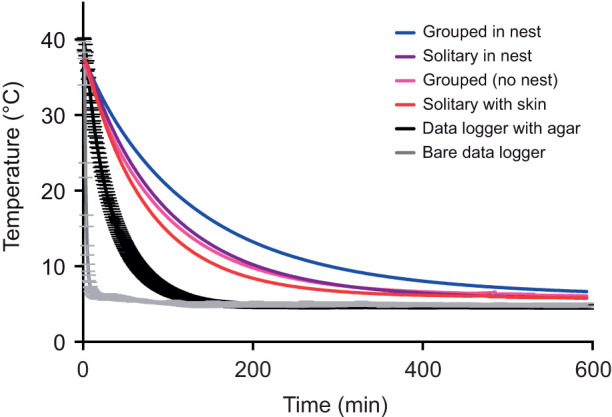
**Cooling curves and experimental treatments.** All curves are plotted.

The infrared thermal images showed how the core of the body maintained the heat (33.8°C, in red) until the fourth hour of the cooling, while maintaining a cooler envelope (skin with fur, in yellow, Fig. 2A–D). These images also show, qualitatively, the better heat conservation of clustered individuals compared with solitary individuals. The adjustment of every one of the 50 cooling curves had an *R*^2^ greater than 0.98 (all curves are plotted in Fig. 3).

In terms of heat conservation, grouped mannequins within nests conserved heat 31% longer (half-time: 91.7±4.71 min; Table 1) than solitary mannequins in nests (half-time: 63.2±2.8 min; Fisher *post hoc* test, *P*=0.008; Table 1). However, there were non-significant differences between half-times of solitary individuals in nest and grouped individuals without a nest (63.2±2.7 versus 64.1±3.6; Fisher *post hoc* test, *P*=0.45; Table 1). Solitary individuals without a nest (half-time: 52.6±3.0 min) conserved heat 18% lower than grouped individuals without a nest (half-time: 64.1±3.6 min; Fisher *post hoc* test, *P*=0.001; Table 1).

**Fig. 4.**
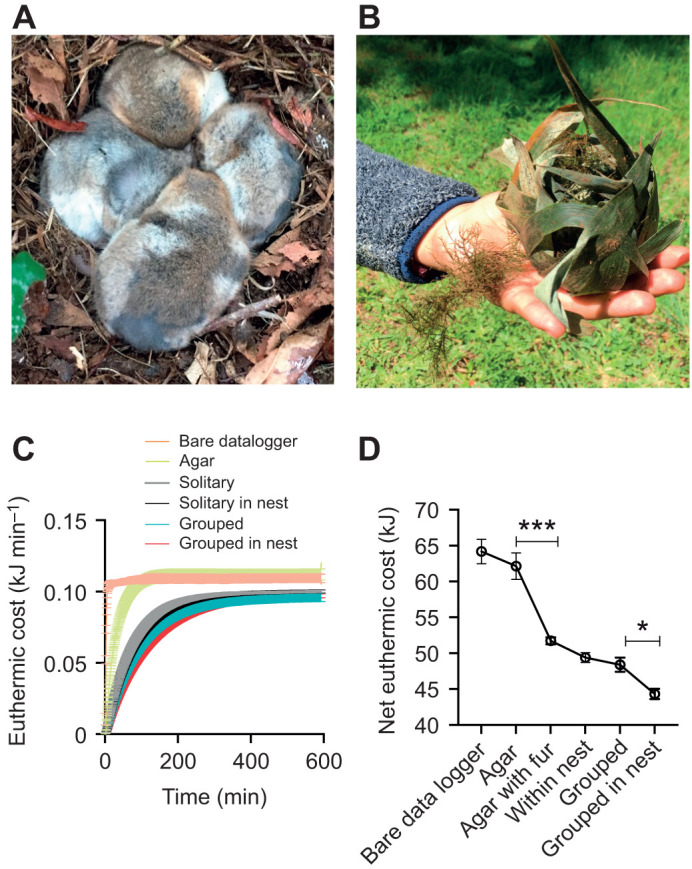
**Heat conservation with clustering and nests.** (A) Clustered (live) individuals in a typical configuration found in tree holes and nests (photo: R. Nespolo). (B) A ‘fresh’ nest found after winter 2020 winter. (C) Euthermic costs of maintenance per minute, calculated during experimental coolings from 35 to 5°C in a climatic chamber (see Materials and Methods for details). This calculation assumes that the animal replaces the heat lost by an equivalent amount of metabolic heat production (Newton's passive cooling). (D) The net euthermic cost of maintenance (mean±s.e.m.) over the complete cooling trial. Asterisks denote significant differences between groups after a one-way ANOVA and Fisher *post hoc* test (*P<0.05; ***P<0.001).

The amount of metabolic heat that a monito should produce every minute to maintain a constant body temperature represents the euthermic cost of maintenance (*E*_cost_). This was calculated using Newton's passive cooling equation (see Materials and Methods) and estimated for clustering mannequins (mimicking a group of monitos, as shown in Fig. 4A) and mannequins within nests (Fig. 4B), and is presented in Fig. 4C. This comparison shows that the configuration that minimizes *E*_cost_ is to be grouped within a nest (red line in Fig. 4C). Summing *E*_cost_ values across the cooling period confirms that the net euthermic cost of maintenance is minimized by the combined use of nest in groups (Fig. 4D). Indeed, significant differences were found after a one-way ANOVA (*F*_5,44_=49.4, *P*=0.0001; Fig. 4D). Specifically, being grouped in the nest has an *E*_cost_ (44.5±0.83 kJ) that is significantly lower than that of being grouped outside a nest (Fisher *post hoc* test, 48.1±1.38 kJ, *P*=0.008). However, being solitary in the nest (50.8±0.62 kJ) is equivalent (not significantly different) to being grouped without a nest (48.1±1.38 kJ; Fisher *post hoc* test, *P*=0.07). There were significant differences in *E*_cost_ between the mannequin with and without fur (51.4±0.55 versus 62.1±1.85 kJ; Fisher *post hoc* test, *P*=0.0001). Finally, the calculated *E*_cost_ of the bare data logger and the mannequin without fur was not significant (64.2±1.70 versus 62.1±1.85 kJ; Fisher *post hoc* test, *P*=0.26).

## DISCUSSION

### Communal nesting, bioenergetics and sociality

There is a historic discussion about whether nest sharing in clusters has a bioenergetic significance (i.e. a heat-conservation strategy) or represents a by-product of the benefits of group living (Dausmann and Glos, 2015; Ebensperger, 2001; Franco et al., 2011; Gilbert et al., 2010; Heenan and Seymour, 2011; Lubbe et al., 2018; Madikiza and San, 2020; Olson et al., 2018; Selonen et al., 2014; Vickery and Millar, 1984; Vogt and Lynch, 1982; Willis and Brigham, 2007; Withers and Jarvis, 1980). The problem arises because often, species with advanced levels of sociality also benefit from clustering during cold periods. For instance, in the woodland dormouse (*Graphiurus murinus*), social clustering seems to be explained by mating behavior rather than by thermoregulatory benefits (Madikiza and San, 2020), and in North American flying squirrels (genus *Glaucomys*), interspecific social nesting appears to be driven by sociality (Olson et al., 2018; Selonen et al., 2014). However, in birds, it has been documented that social thermoregulation combined with the use of insulative roosts and nests provides energy savings of over 50% compared with isolated birds outside nests (Lubbe et al., 2018). This is also the case of Siberian hamsters, which compensate for the reduction in insulation owing to experimental haircuts by using nest materials that were provided experimentally (Kauffman et al., 2003). Thus, in general, grouping and nest use reduce the total conductance of the group, reducing the lower limit of thermoneutrality of the whole cluster (Bozinovic et al., 1988). Therefore, our results, indicating that the optimal strategy is clustering and nest use, are in line with thermodynamic predictions.

### Impact on energy budget

In the field, monitos exhibit high phenotypic flexibility in nest-building behaviour and may build nests within tree cavities or in more exposed locations on tree branches (Vazquez et al., 2020). Also, both the mass and the volume of the nests increase with elevation, thus suggesting that animals build more insulated nests in colder environments (Altamirano et al., 2019). This strategy is combined with huddling behavior, as larger groups are often found in comparatively colder locations (Altamirano et al., 2019; Celis-Diez et al., 2012). Indeed, according to Canals et al. (1997), the average relative surface area reduction during huddling in small mammals ranges from 28.7 to 39.1%, where the maximum reduction in surface area is attained with three individuals. Indeed, according to Bozinovic et al. (1988), the reduction in minimum thermal conductance owing to huddling and nest use is approximately 42% (compared with animals housed individually), and represents an important fraction of the energy budget of a small marsupial or rodent, especially in winter. However, these authors used *n*=5 individuals and experimental temperatures of −10°C, which is outside the natural range for *D. gliroides*. In our experiments (with minimum *T*_A_ of 5°C), the nests provided additional energy savings of approximately 10% (a reduction in *E*_cost_ from 49 to 44 kJ; see Fig. 4D). Considering that the basal metabolic rate of *D. gliroides* is approximately 13 kJ day^−1^ (Nespolo et al., 2022a), these 5 kJ represent almost half of energy consumption per day in a resting, thermoneutral condition.

### Hibernation and communal nesting

In heterothermic species [i.e. animals that express daily or multiday torpor (hibernation), *sensu* Ruf and Geiser, 2015], of which *D. gliroides* is an example (Nespolo et al., 2021), huddling has been associated with increased survival (Boratyński et al., 2015; Boyles et al., 2008; Patil et al., 2013). However, when torpid, animals reduce the set point of temperature regulation, thus promoting heat loss instead of heat conservation, until this set point is attained (Geiser, 2011; Humphries et al., 2002; Mejías et al., 2022; Nespolo et al., 2021). Then, if communal nesting is a strategy for conserving heat, it should be beneficial at relatively high temperatures, where animals promote euthermic thermoregulation (Nespolo et al., 2022a; Nowack and Geiser, 2016). Although several results support the idea that heterothermic species cluster together when euthermic (Bustamante et al., 2002; Gilbert et al., 2010; Jefimow et al., 2011; Nespolo et al., 2022a; Willis and Brigham, 2007; Wojciechowski et al., 2011), extreme hibernators (e.g. animals that hibernate near 0°C, such as arctic ground squirrels, hoary marmots or monitos at high Andean locations) tend to hibernate solitarily (Barnes, 1989; Mejías et al., 2021; Patil et al., 2013).

Recently, an experimental field study conducted on *D. gliroides* conclusively showed that overfed individuals that aborted hibernation formed larger groups and used experimental hibernacula more frequently, compared with those that received less food and hibernated normally (which were found more often outside the hibernacula; see details in Nespolo et al., 2022a). Thus, the ‘torpor-prone’ individuals (i.e. animals with a fall in blood glucose, the main trigger of torpor, see Lo Martire et al., 2018; Westman and Geiser, 2004) search for cold places and avoid huddling to minimize energy expenditure. This has been observed both in *D. gliroides* (Nespolo et al., 2022a) and in hibernating bats, which even experience internal migrations during short arousals, within roosting caves, to select cooler sites (Ryan et al., 2019). On the contrary, individuals reluctant to use torpor (i.e. those that are well fed) promote euthermic thermoregulation by selecting refugia and huddling. This explains the non-significant differences found by Franco et al. (2012) when comparing energy expenditure of clustered and isolated animals in the laboratory: torpid and active animals were mixed in the chamber. These authors concluded that the main response of *D. gliroides* to low ambient temperature was reduced body temperature and torpor, irrespective of huddling. Now we know, from the cited field studies, that this is incorrect.

In summary, the results presented in the present study, together with the cited field experiments performed in *D. gliroides*, in our opinion clarifies the fact that communal nesting (including the crucial isolating effect of the *Dromiciops* nest) is essential to this species during cold periods, and when euthermic.

### Biophysical models, energy budget and conservation

The use of biophysical models in physiological ecology dates back to Porter and Gates's seminar paper (Porter and Gates, 1969; Tracy, 1972; see an update in Kearney and Porter, 2009), which defined the energy budget of animals as a balance between energy losses and gains in a variety of environments, organism types and thermal conditions. Later, a number of authors applied them in different contexts (e.g. climatic impact on species range: Huey et al., 2012; Kearney et al., 2009; heat loss and behavioral thermoregulation in endotherms: Kenagy et al., 2002; McCafferty et al., 2011; reconstructing dinosaur physiology: Seebacher, 2003), and recently, for predicting habitat suitability for mammalian conservation (McComb et al., 2021). Here, we show that simple simulations could provide quantitative data for testing a specific hypothesis about the origin of sociality in a marsupial. Today, *Dromiciops* seems to be the only American marsupial with advanced levels of sociality, in which social groups seem to be maintained in time, above the level of the family (Nespolo et al., 2022b). Our results supporting a net energetic benefit of communal nesting warrant further field studies for analyzing how this social behavior extrapolates in nature.

## References

[JEB244606C1] Altamirano, T. A., Honorato, M. T., Ibarra, J. T., de la Maza, M., de Zwaan, D. R., Bonacic, C. and Martin, K. (2019). Elevation has contrasting effects on avian and mammalian nest traits in the Andean temperate mountains. *Austral. Ecol.* 44, 691-701. 10.1111/aec.12718

[JEB244606C4] Antinuchi, C. D. and Busch, C. (2001). Reproductive energetics and thermoregulatory status of nestlings in pampas mice *Akodon azarae* (Rodentia: Sigmodontinae). *Physiol. Biochem. Zool.* 74, 319-324. 10.1086/320416 11331503

[JEB244606C5] Barnes, B. M. (1989). Freeze avoidance in a mammal: body temperatures below 0°C in an Arctic hibernator. *Science* 244, 1593-1595. 10.1126/science.2740905 2740905

[JEB244606C6] Boratyński, J. S., Willis, C. K. R., Jefimow, M. and Wojciechowski, M. S. (2015). Huddling reduces evaporative water loss in torpid Natterer's bats, *Myotis nattereri*. *Comp. Biochem. Physiol. A Mol. Integr. Physiol.* 179, 125-132. 10.1016/j.cbpa.2014.09.035 25289993

[JEB244606C7] Boyles, J. G., Storm, J. J. and Brack, V. Jr. (2008). Thermal benefits of clustering during hibernation: a field test of competing hypotheses on *Myotis sodalis*. *Funct. Ecol.* 22, 632-636. 10.1111/j.1365-2435.2008.01423.x

[JEB244606C8] Bozinovic, F., Rosenmann, M. and Ruiz, G. (1987). Transferencia de calor, convección y gradiente altitudinal. *Arch. Biol. Med. Exp.* 20, 85-88. 8929091

[JEB244606C9] Bozinovic, F., Rosenmann, M. and Veloso, C. (1988). Termorregulación conductual en *Phyllotis darwini* (Rodentia: Cricetidae): efecto de la temperatura ambiente, uso de nidos y agrupamiento social sobre el gasto de energía. *Rev. Chil. Hist. Nat.* 61, 81-86.

[JEB244606C10] Bozinovic, F., Ruiz, G. and Rosenmann, M. (2004). Energetics and torpor of a South American ‘living fossil’, the microbiotheriid *Dromiciops gliroides*. *J. Comp. Physiol. B Biochem. Syst. Environ. Physiol.* 174, 293-297. 10.1007/s00360-004-0414-8 14760502

[JEB244606C11] Bustamante, D. M., Nespolo, R. F., Rezende, E. L. and Bozinovic, F. (2002). Dynamic thermal balance in the leaf-eared mouse: the interplay among ambient temperature, body size, and behavior. *Physiol. Biochem. Zool.* 75, 396-404. 10.1086/342253 12324896

[JEB244606C12] Canals, M., Rosenmann, M. and Bozinovic, F. (1989). Energetics and geometry of huddling in small mammals. *J. Theor. Biol.* 141, 181-189. 10.1016/S0022-5193(89)80016-5 2632987

[JEB244606C13] Canals, M., Rosenmann, M. and Bozinovic, F. (1997). Geometrical aspects of the energetic effectiveness of huddling in small mammals. *Acta Theriol.* 42, 321-328. 10.4098/AT.arch.97-32

[JEB244606C14] Celis-Diez, J. L., Hetz, J., Marín-Vial, P. A., Fuster, G., Necochea, P., Vásquez, R. A., Jaksic, F. M. and Armesto, J. J. (2012). Population abundance, natural history, and habitat use by the arboreal marsupial *Dromiciops gliroides* in rural Chiloé Island, Chile. *J. Mammal.* 93, 134-148. 10.1644/10-MAMM-A-406.1

[JEB244606C15] Dausmann, K. H. and Glos, J. (2015). No energetic benefits from sociality in tropical hibernation. *Funct. Ecol.* 29, 498-505. 10.1111/1365-2435.12368

[JEB244606C16] Ebensperger, L. A. (2001). A review of the evolutionary causes of rodent group-living. *Acta Theriol.* 46, 115-144. 10.4098/AT.arch.01-16

[JEB244606C17] Ebensperger, L. A. and Bozinovic, F. (2000). Communal burrowing in the hystricognath rodent, *Octodon degus*: a benefit of sociality? *Behav. Ecol. Sociobiol.* 47, 365-369. 10.1007/s002650050678

[JEB244606C18] Ebensperger, L. and Labra, A. (2020). *Comportamiento Social de la Fauna Nativa de Chile*. Ediciones UC.

[JEB244606C19] Ebensperger, L. A., Hurtado, M. J., Soto-Gamboa, M., Lacey, E. A. and Chang, A. T. (2004). Communal nesting and kinship in degus (*Octodon degus*). *Naturwissenschaften* 91, 391-395. 10.1007/s00114-004-0545-5 15309311

[JEB244606C20] Fisher, D. O., Nuske, S., Green, S., Seddon, J. M. and McDonald, B. (2011). The evolution of sociality in small, carnivorous marsupials: the lek hypothesis revisited. *Behav. Ecol. Sociobiol.* 65, 593-605. 10.1007/s00265-010-1060-7

[JEB244606C21] Fonturbel, F. E., Franco, L. M., Bozinovic, F., Quintero-Galvis, J. F., Mejias, C., Amico, G. C., Vázquez, M. S., Sabat, P., Sánchez-Hernández, J. C., Watson, D. M. et al. (2022). The ecology and evolution of the Monito del monte, a relict species from the southern South America temperate forests. *Ecol. Evol.* 12, e8645. 10.1002/ece3.8645 35261741 PMC8888251

[JEB244606C22] Franco, M., Quijano, A. and Soto-Gamboa, M. (2011). Communal nesting, activity patterns, and population characteristics in the near-threatened monito del monte, *Dromiciops gliroides*. *J. Mammal.* 92, 994-1004. 10.1644/10-MAMM-A-256.1

[JEB244606C23] Franco, M., Contreras, C., Cortés, P., Chappell, M. A., Soto-Gamboa, M. and Nespolo, R. F. (2012). Aerobic power, huddling and the efficiency of torpor in the South American marsupial, *Dromiciops gliroides*. *Biol. Open* 1, 1178-1184. 10.1242/bio.20122790 23259051 PMC3522878

[JEB244606C24] Geiser, F. (2011). Hibernation: endotherms. *eLS* 2011, 1-10. 10.1002/9780470015902.a0003215.pub2

[JEB244606C25] Gilbert, C., McCafferty, D., Le Maho, Y., Martrette, J.-M., Giroud, S., Blanc, S. and Ancel, A. (2010). One for all and all for one: the energetic benefits of huddling in endotherms. *Biol. Rev.* 85, 545-569. 10.1111/j.1469-185X.2009.00115.x 20039866

[JEB244606C26] Gurovich, Y., Stannard, H. J. and Old, J. M. (2015). The presence of the marsupial *Dromiciops gliroides* in Parque Nacional Los Alerces, Chubut, Southern Argentina, after the synchronous maturation and flowering of native bamboo and subsequent rodent irruption. *Rev. Chil. Hist. Nat.* 88, 17. 10.1186/s40693-015-0047-1

[JEB244606C27] Heenan, C. B. and Seymour, R. S. (2011). Structural support, not insulation, is the primary driver for avian cup-shaped nest design. *Proc. R. Soc. B Biol. Sci.* 278, 2924-2929. 10.1098/rspb.2010.2798 PMC3151712 21325330

[JEB244606C28] Honorato, M. T., Altamirano, T. A., Ibarra, J. T., De la Maza, M., Bonacic, C. and Martin, K. (2016). Composition and preferences regarding nest materials by cavity-nesting vertebrates in the Andean temperate forest of Chile. *Bosque* 37, 485-492. 10.4067/S0717-92002016000300005

[JEB244606C29] Huey, R. B., Kearney, M. R., Krockenberger, A., Holtum, J. A. M., Jess, M. and Williams, S. E. (2012). Predicting organismal vulnerability to climate warming: roles of behaviour, physiology and adaptation. *Philos. Trans. R. Soc. B Biol. Sci.* 367, 1665-1679. 10.1098/rstb.2012.0005 PMC3350654 22566674

[JEB244606C30] Humphries, M. M., Thomas, D. W. and Speakman, J. R. (2002). Climate-mediated energetic constraints on the distribution of hibernating mammals. *Nature* 418, 313-316. 10.1038/nature00828 12124621

[JEB244606C31] Jefimow, M., Głąbska, M. and Wojciechowski, M. S. (2011). Social thermoregulation and torpor in the Siberian hamster. *J. Exp. Biol.* 214, 1100-1108. 10.1242/jeb.050823 21389194

[JEB244606C32] Kauffman, A. S., Paul, M. J., Butler, M. P. and Zucker, I. (2003). Huddling, locomotor, and nest-building behaviors of furred and furless Siberian hamsters. *Physiol. Behav.* 79, 247-256. 10.1016/S0031-9384(03)00115-X 12834796

[JEB244606C33] Kearney, M. and Porter, W. (2009). Mechanistic niche modelling: combining physiological and spatial data to predict species’ ranges. *Ecol. Lett.* 12, 334-350. 10.1111/j.1461-0248.2008.01277.x 19292794

[JEB244606C34] Kearney, M., Porter, W. P., Williams, C., Ritchie, S. and Hoffmann, A. A. (2009). Integrating biophysical models and evolutionary theory to predict climatic impacts on species’ ranges: the dengue mosquito *Aedes aegypti* in Australia. *Funct. Ecol.* 23, 528-538. 10.1111/j.1365-2435.2008.01538.x

[JEB244606C35] Kenagy, G. J., Nespolo, R. F., Vasquez, R. A. and Bozinovic, F. (2002). Daily and seasonal limits of time and temperature to activity of degus. *Rev. Chil. Hist. Nat.* 75, 567-581. 10.4067/S0716-078X2002000300008

[JEB244606C36] Lo Martire, V., Valli, A., Bingaman, M. J., Zoccoli, G., Silvani, A. and Swoap, S. J. (2018). Changes in blood glucose as a function of body temperature in laboratory mice: implications for daily torpor. *Am. J. Physiol. Endocrinol. Metab.* 315, E662-E670. 10.1152/ajpendo.00201.2018 30040481 PMC6230715

[JEB244606C37] Lubbe, N., Czenze, Z. J., Noakes, M. J. and McKechnie, A. E. (2018). The energetic significance of communal roosting and insulated roost nests in a small arid-zone passerine. *Ostrich* 89, 347-354. 10.2989/00306525.2018.1538061

[JEB244606C38] Madikiza, Z. J. K. and San, E. D. (2020). Patterns of nest box sharing in woodland dormice (*Graphiurus murinus*): Evidence for intra-sexual tolerance and communal nesting. *Behav. Process.* 177, 104141. 10.1016/j.beproc.2020.104141 32445854

[JEB244606C39] McCafferty, D. J., Gilbert, C., Paterson, W., Pomeroy, P. P., Thompson, D., Currie, J. I. and Ancel, A. (2011). Estimating metabolic heat loss in birds and mammals by combining infrared thermography with biophysical modelling. *Comp. Biochem. Physiol. A Mol. Integr. Physiol.* 158, 337-345. 10.1016/j.cbpa.2010.09.012 20869456

[JEB244606C40] McComb, L. B., Lentini, P. E., Harley, D. K. P., Lumsden, L. F., Eyre, A. C. and Briscoe, N. J. (2021). Climate and behaviour influence thermal suitability of artificial hollows for a critically endangered mammal. *Anim. Conserv.* 25, 401-413. 10.1111/acv.12750

[JEB244606C41] Mejías, C., Castro-Pastene, C. A., Carrasco, H., Quintero-Galvis, J. F., Soto-Gamboa, M., Bozinovic, F. and Nespolo, R. F. (2021). Natural history of the relict marsupial Monito del Monte at the most extreme altitudinal and latitudinal location. *Ecosphere* 12, 1-15. 10.1002/ecs2.357734938591

[JEB244606C42] Mejías, C., Navedo, J. G., Sabat, P., Franco, L. M., Bozinovic, F. and Nespolo, R. F. (2022). Body composition and energy savings by hibernation in the South American marsupial *Dromiciops gliroides*: a field study applying quantitative magnetic resonance. *Physiol. Biochem. Zool.* 95, 239-250. 35443149 10.1086/719932

[JEB244606C43] Mitchell, K. J., Pratt, R. C., Watson, L. N., Gibb, G. C., Llamas, B., Kasper, M., Edson, J., Hopwood, B., Male, D., Armstrong, K. N. et al. (2014). Molecular phylogeny, biogeography and habitat preference evolution of marsupials. *Mol. Biol. Evol.* 31, 2322-2330. 10.1093/molbev/msu176 24881050

[JEB244606C44] Nespolo, R. F., Opazo, J. C., Rosenmann, M. and Bozinovic, F. (1999). Thermal acclimation, maximum metabolic rate, and nonshivering thermogenesis of *Phyllotis xanthopygus* (Rodentia) in the Andes mountains. *J. Mammal.* 80, 742-748. 10.2307/1383243

[JEB244606C45] Nespolo, R. F., Mejias, C., Espinoza, A., Quintero-Galvis, J. F., Rezende, E. L., Fonturbel, F. E. and Bozinovic, F. (2021). Heterothermy as the norm, homeothermy as the exception: variable torpor patterns in the South American marsupial monito del monte (*Dromiciops gliroides*). *Front. Physiol.* 12, 682394. 10.3389/fphys.2021.682394 34322034 PMC8311349

[JEB244606C46] Nespolo, R. F., Fontúrbel, F. E., Mejias, C., Contreras, R., Gutierrez, P., Oda, E., Sabat, P., Hambly, C., Speakman, J. R. and Bozinovic, F. (2022a). A Mesocosm experiment in ecological physiology: the modulation of energy budget in a hibernating marsupial under chronic caloric restriction. *Physiol. Biochem. Zool.* 95, 66-81. 10.1086/717760 34875208

[JEB244606C47] Nespolo, R. F., Saenz-Agudelo, P., Mejias, C., Quintero-Galvis, J. F., Peña, I., Sabat, P., Sanchez-Hernandez, J. C. and Gurovich, Y. (2022b). The physiological ecology of the enigmatic colocolo opossum, the monito del monte (genus *Dromiciops*) and its role as a bioindicator of the broadleaf biome. In *Marsupials as Bioindicators* (ed. M. L. Larramendy), pp. 81-112. London: Royal Society of Chemistry.

[JEB244606C48] Nicol, S. C. and Andersen, N. A. (2007). Cooling rates and body temperature regulation of hibernating echidnas (*Tachyglossus aculeatus*). *J. Exp. Biol.* 210, 586-592. 10.1242/jeb.02701 17267644

[JEB244606C49] Nowack, J. and Geiser, F. (2016). Friends with benefits: the role of huddling in mixed groups of torpid and normothermic animals. *J. Exp. Biol.* 219, 590-596. 10.1242/jeb.128926 26685170

[JEB244606C50] Oda, E., Rodríguez-Gómez, G. B., Fontúrbel, F. E., Soto-Gamboa, M. and Nespolo, R. F. (2019). Southernmost records of *Dromiciops gliroides*: extending its distribution beyond the Valdivian rainforest. *Gayana* 83, 145-149. 10.4067/S0717-65382019000200145

[JEB244606C51] Olson, M. N., Bowman, J. and Burness, G. (2018). Social thermoregulation does not explain heterospecific nesting in North American flying squirrels. *Biol. J. Linn. Soc.* 123, 805-813. 10.1093/biolinnean/bly014

[JEB244606C52] Patil, V. P., Morrison, S. F., Karels, T. J. and Hik, D. S. (2013). Winter weather versus group thermoregulation: what determines survival in hibernating mammals? *Oecologia* 173, 139-149. 10.1007/s00442-013-2612-0 23456241

[JEB244606C53] Porter, W. P. and Gates, D. M. (1969). Thermodynamic equilibria of animals with environment. *Ecol. Monogr.* 39, 227-244. 10.2307/1948545

[JEB244606C54] Rezende, E. L. and Bacigalupe, L. D. (2015). Thermoregulation in endotherms: physiological principles and ecological consequences. *J. Comp. Physiol. B Biochem. Syst. Environ. Physiol.* 185, 709-727. 10.1007/s00360-015-0909-5 26025431

[JEB244606C55] Rough, J. P. and Bharathan, D. (2005). Predicting human thermal comfort in automobiles. Vehicle Thermal Management Systems Conference. Toronto: SAE International.

[JEB244606C56] Ruf, T. and Geiser, F. (2015). Daily torpor and hibernation in birds and mammals. *Biol. Rev.* 90, 891-926. 10.1111/brv.12137 25123049 PMC4351926

[JEB244606C57] Russell, E. M. (1984). Social-behavior and social-organization of marsupials. *Mammal. Rev.* 14, 101-154. 10.1111/j.1365-2907.1984.tb00343.x

[JEB244606C58] Ryan, C. C., Burns, L. E. and Broders, H. G. (2019). Changes in underground roosting patterns to optimize energy conservation in hibernating bats. *Can. J. Zool.* 97, 1064-1070. 10.1139/cjz-2018-0340

[JEB244606C59] Scholander, P. F. (1955). Evolution of climatic adaptations in homeotherms. *Evolution* 9, 15-26. 10.1111/j.1558-5646.1955.tb01510.x

[JEB244606C60] Seebacher, F. (2003). Dinosaur body temperatures: the occurrence of endothermy and ectothermy. *Paleobiology* 29, 105-122. 10.1666/0094-8373(2003)029<0105:DBTTOO>2.0.CO;2

[JEB244606C61] Selonen, V., Hanski, I. K. and Wistbacka, R. (2014). Communal nesting is explained by subsequent mating rather than kinship or thermoregulation in the Siberian flying squirrel. *Behav. Ecol. Sociobiol.* 68, 971-980. 10.1007/s00265-014-1709-8

[JEB244606C62] Sharbaugh, S. M. (2001). Seasonal acclimatization to extreme climatic conditions by black-capped chickadees (*Poecile atricapilla*) in interior Alaska (64°N). *Physiol. Biochem. Zool.* 74, 568-575. 10.1086/322170 11436141

[JEB244606C63] Tracy, C. R. (1972). Newotn's law: its applicability for expression heat losses from homeotherms. *Bioscience* 22, 656-659. 10.2307/1296267

[JEB244606C64] Vasquez, M. S., Rodríguez-Cabal, M. A., Gonzalez, D. V., Pacheco, G. S. and Amico, G. C. (2018). Different nest predator guild associated to egg size in the Patagonian temperate forest. *Bird Study* 65, 478-483. 10.1080/00063657.2018.1555572

[JEB244606C65] Vazquez, M. S., Ibarra, J. T. and Altamirano, T. A. (2020). Austral opossum adjusts to life in second-growth forests by nesting outside cavities. *Austral. Ecol.* 45, 1179-1182. 10.1111/aec.12927

[JEB244606C66] Vickery, W. L. and Millar, J. S. (1984). The energetics of huddling by endotherms. *Oikos* 43, 88-93. 10.2307/3544249

[JEB244606C67] Vogt, F. D. and Lynch, G. R. (1982). Influence of ambient temperature, nest availability, huddling, and daily torpor on energy expenditure in the white-footed mouse *Peromyscus leucopus*. *Physiol. Zool.* 55, 56-63. 10.1086/physzool.55.1.30158443

[JEB244606C68] Westman, W. and Geiser, F. (2004). The effect of metabolic fuel availability on thermoregulation and torpor in a marsupial hibernator. *J. Comp. Physiol. B Biochem. Syst. Environ. Physiol.* 174, 49-57. 10.1007/s00360-003-0388-y 14513266

[JEB244606C69] Willis, C. K. R. and Brigham, R. M. (2007). Social thermoregulation exerts more influence than microclimate on forest roost preferences by a cavity-dwelling bat. *Behav. Ecol. Sociobiol.* 62, 97-108. 10.1007/s00265-007-0442-y

[JEB244606C70] Withers, P. C. and Jarvis, J. U. M. (1980). The effect of huddling on thermoregulation and oxygen consumption for the naked mole-rat. *Comp. Biochem. Physiol. A* 66A, 215-219. 10.1016/0300-9629(80)90154-1

[JEB244606C71] Wojciechowski, M. S., Jefimow, M. and Pinshow, B. (2011). Heterothermy, and the energetic consequences of huddling in small migrating passerine birds. *Integr. Comp. Biol.* 51, 409-418. 10.1093/icb/icr055 21693540

[JEB244606C72] Zhang, M., Che, Z. H., Chen, J. H., Zhao, H. Z., Yang, L., Zhong, Z. Y. and Lu, J. H. (2011). Experimental determination of thermal conductivity of water-agar gel at different concentrations and temperatures. *J. Chem. Eng. Data* 56, 859-864. 10.1021/je100570h

